# Magnetic Resonance Imaging in Marchiafava-Bignami Syndrome: A Cornerstone in Diagnosis and Prognosis

**DOI:** 10.1155/2014/609708

**Published:** 2014-09-23

**Authors:** Kathyayini Paidipati Gopalkishna Murthy

**Affiliations:** Department of Radiology, American Oncology Institute and Citizens Hospitals, Serilingampally, Hyderabad, Telangana 500019, India

## Abstract

Marchiafava-Bignami syndrome is a rare condition. However, with the advent of MRI, more and more of these cases are being diagnosed. Thus, it becomes essential for a radiologist to be familiar with its imaging features as well as clinical presentation. A 50-year-old chronic alcoholic presented to the emergency room with history of 3 episodes of seizures 2 days earlier. The patient had gait disturbances for the last few days. On examination, the patient was in a state of stupor. No neck rigidity was elicited. On MRI, the corpus callosum appeared diffusely hypointense on T1 weighted images and hyperintense on T2 weighted sequences without any evident enhancement after intravenous administration of Gadolinium. On fluid attenuation and inversion recovery images, central hypointensity with surrounding hyperintense rim involving the genu, body, and splenium of corpus callosum was noted. Additionally, cortical-subcortical signal intensity changes were also noted predominantly involving the right frontal lobe. On diffusion weighted imaging, all the above mentioned lesions showed restriction of diffusion. I am presenting here a case of Marchiafava-Bignami syndrome highlighting the role of MR imaging in diagnosing, prognosticating as well as in understanding the underlying pathophysiology of this rare clinical entity.

## 1. Introduction

Marchiafava-Bignami syndrome (MBS) is a rare demyelinating disorder seen in chronic alcoholics that is characterized by corpus callosal necrosis and associated white matter changes [1].

I present the MRI features of MBS with an attempt to correlate them with the underlying pathophysiology. This report also highlights the role of imaging in predicting prognosis of the disease.

## 2. Case Description

### 2.1. Clinical History

A 50-year-old male chronic alcoholic presented to the emergency room with history of 3 episodes of seizures 2 days earlier. No h/o fever was present. On further enquiry, history of gait disturbances was elicited.

On examination the patient was in a state of stupor. No neck rigidity was elicited.

*Lab investigations *revealed low serum glucose and vitamin B12 levels. CSF examination was normal.

The above observations prompted further imaging evaluation with MRI.

*MR imaging* was performed on a 1.5 T magnet (GE Signa, 1.5 T, General Electric, Milwaukee, Wisconsin). Fast spin-echo T1 weighted (TR: 650 ms, TE: 14 ms), T2 weighted (dual echo TR: 2014 ms; TE: 30 and 100 ms), and fluid attenuation and inversion recovery (FLAIR) (Turbo Spin Echo, turbo factor 11; TR: 5496 ms; TE: 100 ms; inversion time: 2000 ms) images in axial and sagittal planes were acquired. Diffusion weighted imaging (DWI) scans were acquired with diffusion gradients along each of the three principal axes with 3 different *b* values (0, 187, and 757 s/mm^2^). Post-contrast T1 weighted (TR: 650 ms, TE: 14 ms) images were acquired after intravenous administration of 0.2 mL/kg body weight of gadodiamide (Omniscan; Nycomed-Amersham, Oslo, Norway) at a rate of 4 mL/second with a delay of 10 minutes in the axial and sagittal planes.

### 2.2. Imaging Findings

**Figure 1:**
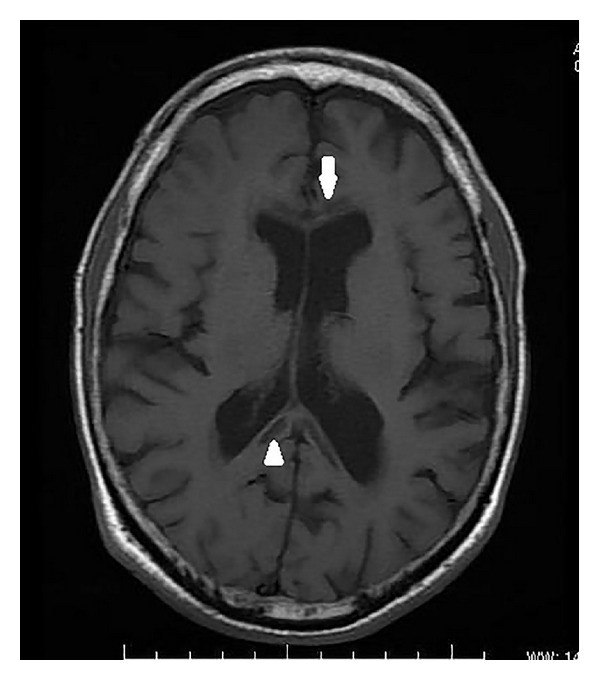
Axial T1 weighted image showing hypointensity of the genu (white arrow) and splenium (white arrowhead) of corpus callosum.

**Figure 2:**
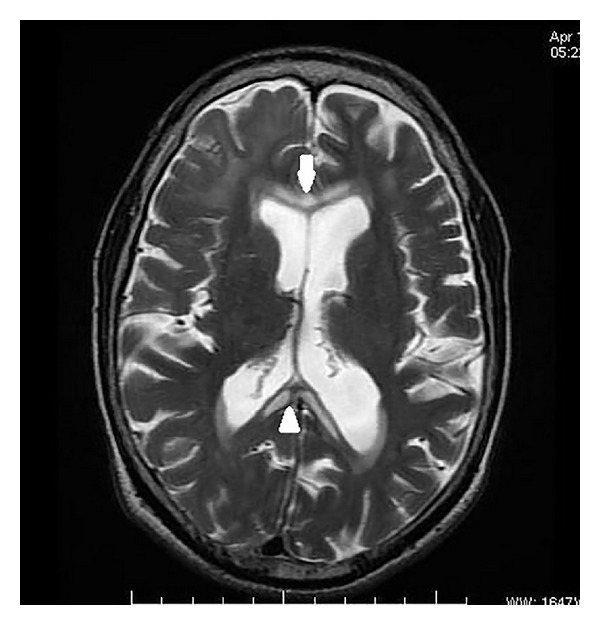
Axial T2 weighted image showing hyperintensity of genu (white arrow) and splenium (white arrowhead) of corpus callosum.

**Figure 3:**
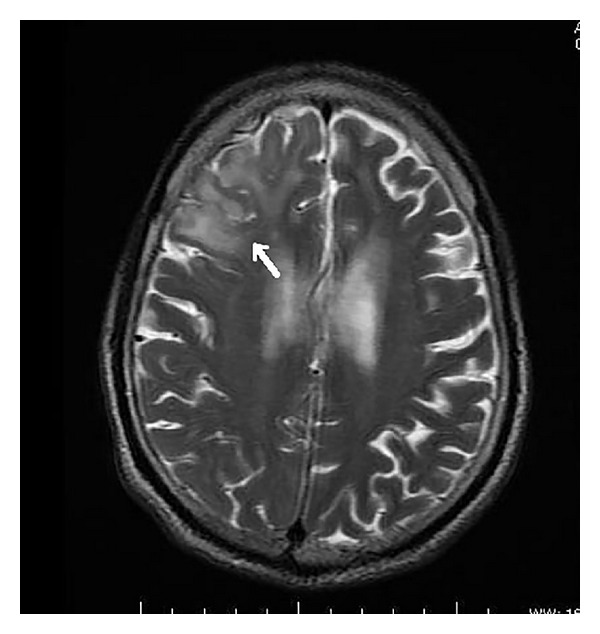
Axial T2 weighted image revealing cortical-subcortical hyperintensity involving the right frontal lobe (white arrow).

**Figure 4:**
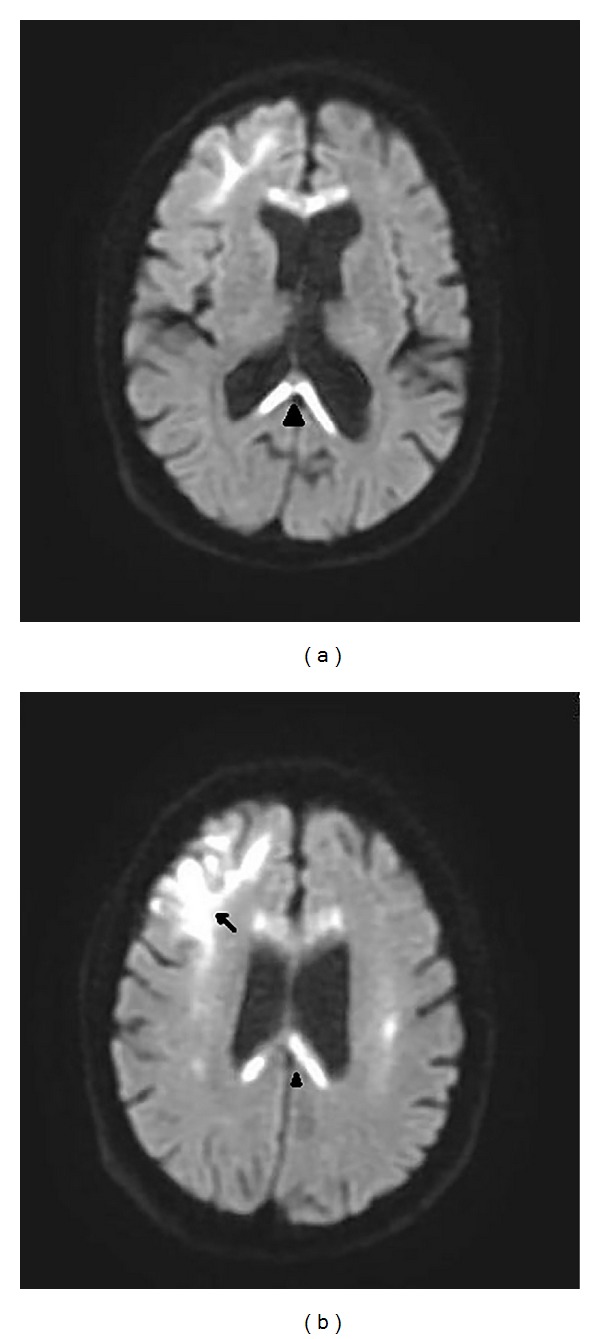
Diffusion weighted images displaying restricted diffusion involving the subcortical right frontal white matter (black arrow) and genu and splenium (black arrowhead) of corpus callosum.

**Figure 5:**
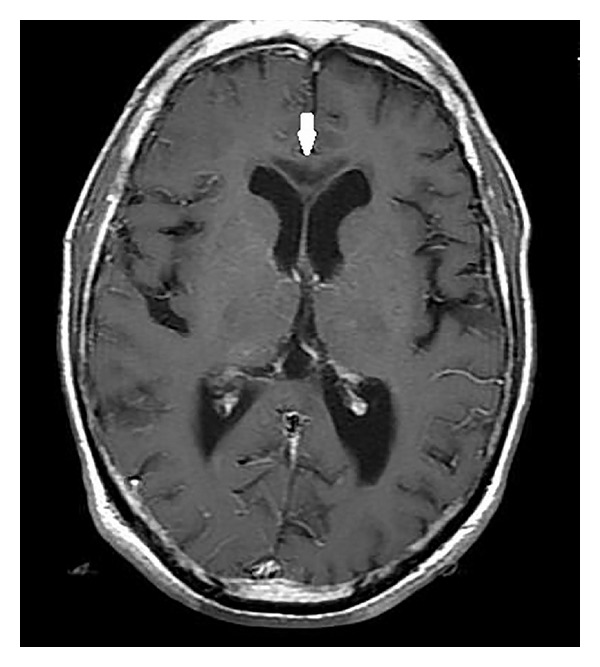
Post-Gadolinium axial T1 weighted image showing lack of enhancement of the hypointense lesions involving the corpus callosum (white arrow).

**Figure 6:**
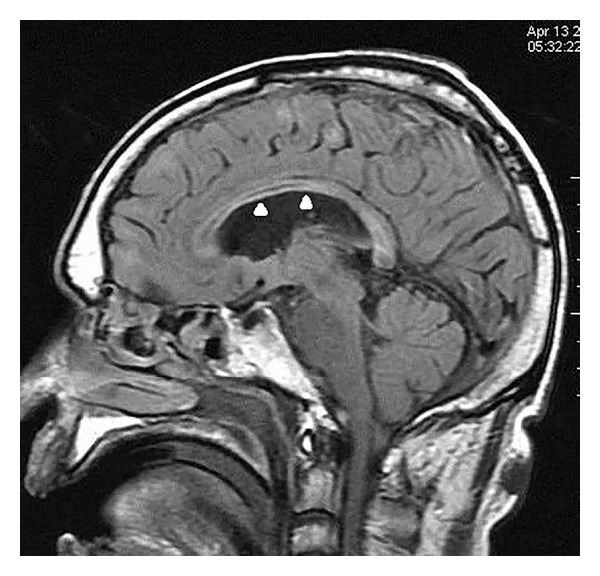
Sagittal fluid attenuation and inversion recovery image displaying central hypointensity (suggesting cavitation) with surrounding hyperintense rim (active inflammation) (white arrowheads) involving the genu, body, and splenium of corpus callosum.

On MRI, the corpus callosum appeared diffusely hypointense on T1WI (Figure 1) and hyperintense on T2WI (Figure 2) without any evident enhancement after intravenous administration of Gadolinium (Figure 5). On FLAIR images, central hypointensity with surrounding hyperintense rim involving the genu, body, and splenium of corpus callosum was noted (Figure 6). Additionally, cortical-subcortical signal intensity changes were also noted predominantly involving the right frontal lobe (Figure 3) appearing hypointense on T1WI and hyperintense on T2WI. On diffusion weighted imaging, all the above mentioned lesions showed restriction of diffusion (Figures 4(a) and 4(b)).

On the basis of the clinical features and imaging appearances, a diagnosis of Marchiafava-Bignami syndrome was made.

### 2.3. Clinical Outcome

The patient was admitted to the neurointensive care unit and succumbed 2 days later despite extensive supportive management.

## 3. Discussion

Marchiafava-Bignami syndrome is characterized by corpus callosum necrosis and is observed predominantly in alcoholics. It usually affects the body of the corpus callosum, followed by the genu and splenium [2]. Lesions may also be found in the hemispheric white matter. Infrequently, patients with MBS can also have clinical and imaging features of central pontine myelinolysis [3].

MBS can present as two main clinical forms: an acute form with severe disturbance of consciousness and neurocognitive deficits, often fatal, and a chronic form which usually presents as chronic dementia. The most important differential diagnosis considered in the current clinical context (acute presentation) was Wernicke's encephalopathy. However, absence of typical clinical signs like ophthalmoplegia, nystagmus, and ataxia along with lack of typical MRI features of Wernicke's encephalopathy such as involvement of mammillary bodies and periaqueductal grey matter excluded this differential. Both of these entities can sometimes coexist and happen to fall under the broad category of alcohol-related encephalopathies. The latter comprise a wide spectrum of CNS derangements caused by chronic alcohol intake, mediated through inflammation, DNA damage, and oxidative stress, sometimes exacerbated by accompanying thiamine deficiency (Wernicke's encephalopathy) and altered plasma osmolality [4]. The chronic form, on the other hand, needs to be distinguished from other forms of dementia like Alzheimer's disease, vascular dementia, and frontotemporal lobar degeneration [1] (see Table 1).

The usual chronology of pathological events in MBS is inflammation (acute phase) followed by demyelination and eventually necrosis and axonal loss (chronic phase) [1]. MRI findings can reflect the underlying pathophysiology responsible for the morphological manifestations (see Table 2).

On MR imaging, patients with MBS show areas of low T1 signal intensity and high T2 and FLAIR signal intensity in the body of the corpus callosum with or without associated lesions in the cerebral white matter. These lesions are devoid of mass effect and may show peripheral contrast enhancement during the acute phase [1]. Although the callosal lesions have been described as the hallmark of this disease, few cases of MBS also demonstrate signal intensity abnormalities in the cerebral parenchyma. Most common areas to be affected are the lateral frontal (as in this case) and the temporal lobes. These areas of signal abnormalities represent glial cells replacing degenerated neurons. This phenomenon has been described by Morel as cortical laminar sclerosis [5]. Cortical involvement in MBS has been described in a few radiological reports. Johkura et al. reported cases of MBS with bilaterally symmetrical lesions in the cerebral cortex, particularly frontal lobes. These cortical lesions were hyperintense on T2WI and FLAIR images and displayed restricted diffusion (markedly increased signal intensity on diffusion weighted images and decreased ADC values on ADC maps). They explained that this may reflect cytotoxic edema which represents the acute phase of Morel's laminar sclerosis [6]. Similarly, Ménégon et al. suggested that a combination of restricted diffusion involving the entire corpus callosum diffusely along with cortical involvement was a harbinger of a poor outcome both for survival and for cognitive recovery [7].

Hence diffuse corpus callosal involvement, presence of cortical involvement, and diffusion restriction, all of which were seen in this case, can be considered as poor prognostic factors while predicting the outcome of MBS.

## 4. Conclusion

Magnetic resonance imaging is a valuable tool in diagnosing, prognosticating as well as in understanding the underlying pathophysiology of Marchiafava-Bignami syndrome.

## Figures and Tables

**Table 1:** Clinical forms of MBS and their differentials [1].

Clinical form of MBS	Predominant symptoms	Clinical differentials and their MRI findings
Acute MBS	Mental confusion, disorientation, neurocognitive deficits, and seizures	(1) **Wernicke encephalopathy** Symmetric involvement of medial thalami, mammillary bodies, tectal plate, and periaqueductal grey matter; hypointense on T1WI; hyperintense on T2W and FLAIR images. Few cases with cortical involvement restricted to motor and premotor areas.

Chronic MBS	Chronic dementia	(1) **Alzheimer disease** Extreme hippocampal and medial temporal lobe atrophy, severe global atrophy in end-stage disease. (2) **Multi-infarct dementia** (i) Diffuse white matter disease with large confluent lesions (hypointense on T1WI and hyperintense on T2W and FLAIR images) affecting at least 25% of white matter, mainly in the periventricular regions. (ii) Multiple lacunar infarcts in frontal white matter, thalami, and basal ganglia. (iii) Strategic infarcts: involving Parieto-temporo-occipital association areas, angular gyrus (middle cerebral artery territory), paramedian thalamic, and inferior medial temporal lobe (posterior cerebral artery territory). (3) **Frontotemporal lobar degeneration (Pick's) disease:** Pronounced atrophy of frontal and/or temporal lobes.

**Table 2:** Correlation of imaging findings in MBS with pathophysiology [1, 6].

	MRI findings	Underlying pathophysiology
1	Hyperintensity on T2 weighted images	Edema and myelin damage
2	Hypointensity on T1 weighted images	Total loss of myelin with replacement of the region by a cyst
3	Hyperintense rims and hypointense cores on FLAIR images	Damage to the myelin at the rim with a central necrotic area
4	Uniformly hyperintense lesions on FLAIR	Mixture of demyelination and edema
5	Areas of restricted diffusion on DWI (acute phase)	Cytotoxic edema
